# Editorial: Axon Neurobiology: Fine-Scale Dynamics of Microstructure and Function

**DOI:** 10.3389/fncel.2020.594361

**Published:** 2020-09-23

**Authors:** Haruyuki Kamiya, Dominique Debanne

**Affiliations:** ^1^Department of Neurobiology, Graduate School of Medicine, Hokkaido University, Sapporo, Japan; ^2^Unité de Neurobiologie des canaux Ioniques et de la Synapse, UMR1072, INSERM, Aix-Marseille Université, Marseille, France

**Keywords:** action potential, axon, excitability, modeling, subcellular recording

The axon has been considered as a high-fidelity digital cable that reliably conducts action potentials toward the presynaptic terminals (Rama et al., 2018). Comprehensive understanding of cell biology and physiology of the axons is the key step in a bottom-up approach in cellular neuroscience, although the small structure of the axon, as well as ultrafast signaling by action potentials, have made the experimental analysis extremely difficult (Ohura and Kamiya, 2016). In this Research Topic, we aimed at illuminating recent advances in the study of axon neurobiology, with a focus on the cell biology of the axon initial segment (AIS), electrophysiology and modeling of axon excitability and transmitter release. These studies highlighted that axonal spike signaling is regulated much dynamically than previously thought, and substantially involved in fine-tuning of neuronal information transfer both in time and space.

Action potentials are generated at the AIS or in the proximal axon near the soma, and therefore the excitability of the AIS is the critical determinant of encoding output trains of the action potential. In a series of papers, this Research Topic gained insight into our understanding of the AIS structure and function. Raghuram et al. analyzed the AIS length and distance from the soma in mice retinal ganglion cells and attempted to correlate AIS length with cell size, and shown that both parameters are linked. Computational modeling suggested that this scaling adjusts the spiking threshold, spike rate to support the light responses unrelated to the cell size of the ganglion cells. A study by Kim et al. addressed an important aspect of auditory experience-dependent AIS structure plasticity of the medial nucleus of trapezoid body (MNTB) neurons, a relay of the ascending auditory pathway, during development and aging. It was shown that AIS length and distance from the soma change with age and auditory experience, and that these structural changes account for the modifications in the excitability of MNTB neurons, as supported by modeling. The paper by Zhang et al. examined the roles of purinergic signaling in the development and maintenance of AIS. Using mouse cultured hippocampal neurons, they demonstrated that P_2_Y_1_ purinergic receptors determine the initial development of AIS structure and function. On the other hand, P_2_X_7_ receptors are involved in the maintenance of AIS maturation. The study by Alpizar et al. explored the roles of the cell adhesion molecule neurofascin-186 (NF-186) in the arrangement of ankyrin G and sodium channels in the AIS. Ablation of NF-186 perturbed ankyrin G accumulation at the AIS and altered expression of sodium channels and therefore suggested the possible contribution of this cell adhesion molecule in AIS-specific molecular organization. Schlüter et al. reported the maturation of AIS in retinal ganglion cells with a focus on the cisternal organelle, a presumed Ca^2+^-store of the AIS, using the marker synaptopodin. Since retinal development, as well as visual deprivation, alter the AIS and synaptopodin distribution, the authors suggested the activity-dependent structural plasticity occur in both retinal ganglion cells and pyramidal cells in the visual cortex.

This Research Topic puts special emphasis on the understanding of basic mechanisms underlying fine-tuning of the excitability of the axons. In this line, a few articles nicely reviewed and summarized the current understandings of the functional significance of the excitability tuning of the axons as well as the underlying molecular identities. In combination with the historical views as well as the recent updates on the biophysical properties of axons, Alcami and El Hady offered a comprehensive review of the computational abilities of axons and the dynamic control of generation and propagation of action potentials. This paper also lights up the issues to be investigated in future studies with cutting-edge technologies. The authors suggested the importance of “hybrid computation” of analog and digital signals and their interplay. Burke and Bender reviewed the mechanisms as well as the functional significance of modulation of ionic channels in axons. Action potential generation and propagation, as well as neurotransmitter release and its short-term plasticity, are critically and dynamically regulated by modulation of ion channels in axonal membranes. The future perspective of looking for the functional consequence of modulation of axonal excitability *in vivo* is of extreme interest. Liu and Rasband provided a nice overview of the axonal spectrin, a key cytoskeletal molecule determining the microstructure of the axons. Thanks to the recent development of super-resolution microscopy, the periodic spatial organization of ring-like structures of actin and subcellular arrangement of spectrin implicate the pivotal role of actin-spectrin cytoskeletons in determining axonal excitable domains such as AIS and nodes of Ranvier. Bonetto et al. reported the early steps of compartmentalization of Kv1 channels and associated molecules preceding myelination of axons in cultured hippocampal GABAergic neurons. Although K^+^ channels are well-known to localized at nodes and juxtaparanodes to secure spike propagation along myelinated axons, they demonstrated that Kv1.2 channels are highly expressed all along the axons and the AIS before myelination, in contrast with “pre-nodes” localization of Na_V1_ channels. Rozov et al. provided an overview of the current understanding of the underlying mechanisms for asynchronous transmitter release from the axon terminals. Ca^2+^ sensors, Ca^2+^ sources, and Ca^2+^ extrusion mechanisms may coordinate to limit the prolonged time course of the asynchronous release. Although high-affinity Ca^2+^ sensor Syt7 has been suggested to play key roles in the asynchronous release, the contribution of other synaptotagmins as well as the Na^+^/Ca^2+^ exchanger (NCX) which determine the rate of Ca^2+^ extrusion is needed to be determined in future investigation. Goaillard et al. summarized the roles of dendrite geometry in the output of action potentials and transmitter release. The authors overviewed the examples of “non-canonical polarity neurons” and even axon-less neurons. They also discussed the case of dorsal root ganglion neurons as an example of unipolar neurons. The diversity of axonal and dendritic roles in shaping neuronal output is of importance for the understanding of neuronal functions.

Several studies also illustrate novel experimental methods to overcome the limitation of previous approaches. Emmenegger et al. provided a nice and concise overview of the methods for studying the propagation of action potential along the axons, i.e., subcellular patch-clamping from the axon, genetically encoded voltage imaging, and CMOS technology-based high-density microelectrode arrays (HD-MEAs). With high temporal resolution and spatial information obtained by recordings with HD-MEAs covering the entire course of axon arbors of cultured hippocampal neurons, the authors focused on fundamental studies on action potential propagation. Bullmann et al. introduced a novel approach for high-throughput scanning of axonal arbors and mapping of axonal delays using cultured cortex neurons grown on HD-MEAs. With a high temporal and spatial resolution of HD-MEA recordings of axonal spikes, they provided the evaluation of axon segmentation and enabled high-throughput functional mapping of axon arbors. Rama et al. adopted simultaneous imaging of presynaptic Ca^2+^ entry and glutamate release at the hippocampal mossy fiber boutons using biolistic transfection of glutamate sensor SF-iGluSnFR and introduction of Ca^2+^ indicator Cal-590 in granule cells from organotypic hippocampal cultures. This technique offers a unique opportunity to evaluate the relationship between presynaptic Ca^2+^ and glutamate release across multiple release sites within the individual presynaptic terminals. With this multiplexed imaging, they demonstrated the evidence for the existence of distinct release sites of glutamate at the mossy fiber synapse. Nagendran and Taylor investigated the intrinsic injury mechanisms following axotomy. Using cultured hippocampal neurons grown on microfluidic chambers, they revealed that Na^+^ influx and reversal of NCX induce dendritic spine loss. The authors also report that Ca^2+^ release from the axonal endoplasmic reticulum (ER) plays a critical role in trans-synaptic hyperexcitability following axotomy.

This Research Topic also highlighted the advantage of modeling approach due to the limitation of the experimental approach in axon physiology. Since the information based on the experimental findings is limited for quantitative evaluation, a simulation approach based on a simple assumption might help in testing the quantitative validity of the hypothesis. Using computer modeling, Zbili and Debanne addressed the contribution of myelination on length constant of analog-digital facilitations (ADFs), a graded modulation of transmitter release due to subthreshold depolarization of axonal membranes. This study demonstrated that myelination enhances the axonal length constant thus suggesting a possible functional significance of ADFs in myelinated axons. An important notion is that the enhanced spatial extent of ADFs with myelination may enhance the contribution of ADFs in fine-tuning of neuronal information processing. An advantage of the modeling approach was further highlighted by the review by Kamiya which focused on the mechanism underlying afterdepolarization that follows axonal action potentials. Using a realistic model of hippocampal mossy fibers in combination with a direct recording from the axon terminals, the author clearly shows the substantial contribution of a capacitive component in axonal afterdepolarization. This study also suggests that voltage-dependent K^+^ and Na^+^ conductance play a critical role in shaping time course of afterdepolarization lasting for several tens of ms. Holland et al. discussed alternative solutions for the famous Hodgkin-Huxley (HH) model of the action potential that entirely lies on the electrical nature of the nerve impulse propagating along the axon. Non-electrical factors such as the mechanical and/or thermal changes are taking into account for an attempt to propose a unified model that takes into account the mechanical wave by the pressure pulse of axoplasm and represents the process of nerve impulse propagation accurately. This paper gave insight into the fundamental understanding of the neve impulse propagating axon. Daur et al. addressed the interaction of different spike initiation sites of proximal and distal axons of the unmyelinated motor axon of the lobster with the simultaneous recordings from the two sites of the same axons. The author points to the fact that centrally generated bursts occurring in the proximal axons and peripheral “ectopic” spike initiation from distal axon show mutually suppressive influence. Although the functional significance of ectopically generated action potentials is not fully understood, this implies that they play an important role in shaping the output of the neuronal network. It is also noteworthy that these rules can be generalized to understanding axonal information processing in the mammalian central nervous system.

As overviewed above, this Research Topics illustrated the current state of knowledge on axon neurobiology, of fundamental importance for the bottom-up approach in the understanding of brain functions. It is also obvious that our continuing efforts to understand axon function will require a combination of cutting edge optical techniques for imaging fine structures with super-resolution technologies (Chéreau et al., 2017), direct electrophysiological recordings from the axon (Kawaguchi and Sakaba, 2015), optogenetic tools (Kim et al., 2017), calcium imaging (Hanemaaijer et al., 2020; Zbili et al., 2020), and computer modeling analysis (Goethals and Brette, 2020).

## Author Contributions

All authors listed have made a substantial, direct and intellectual contribution to the work, and approved it for publication.

## Conflict of Interest

The authors declare that the research was conducted in the absence of any commercial or financial relationships that could be construed as a potential conflict of interest.
